# Antioxidant and Neuroprotective Capacity of Resveratrol-Loaded Polymeric Micelles in In Vitro and In Vivo Models with Generated Oxidative Stress

**DOI:** 10.3390/biomedicines14010063

**Published:** 2025-12-27

**Authors:** Maria Lazarova, Elina Tsvetanova, Almira Georgieva, Miroslava Stefanova, Krasimira Tasheva, Lyubomira Radeva, Magdalena Kondeva-Burdina, Krassimira Yoncheva

**Affiliations:** 1 Institute of Neurobiology, Bulgarian Academy of Sciences, 1113 Sofia, Bulgaria; m.lazarova@gmail.com (M.L.); almirageorgieva@gmail.com (A.G.); mira_stefanova@mail.bg (M.S.); 2 Institute of Plant Physiology and Genetics, Bulgarian Academy of Sciences, 1113 Sofia, Bulgaria; krasitasheva@abv.bg; 3 Faculty of Pharmacy, Medical University of Sofia, 1000 Sofia, Bulgaria; l.radeva@pharmfac.mu-sofia.bg (L.R.); mkondeva@pharmac.mu-sofia.bg (M.K.-B.); kyoncheva@pharmfac.mu-sofia.bg (K.Y.)

**Keywords:** resveratrol, micelles, antioxidant properties, neurotoxicity, neurodegeneration

## Abstract

**Background**: Resveratrol (3,5,4′-trihydroxy-trans-stilbene, RVT) is one of the most extensively studied natural polyphenols, with numerous health benefits documented in the literature. One of its most characterized biological properties is the strong antioxidant capacity. However, its poor biopharmaceutical properties limit its in vivo applicability. In this study, we conducted a detailed comparative analysis of the antioxidant and protective capacity of pure and loaded into Pluronic micelles resveratrol. **Methods**: Various in vitro antioxidant assays, such as DPPH, ABTS, superoxide anion radical scavenging, ferric (FRAP), and copper-reducing power assay (CUPPRAC), and iron-induced lipid peroxidation were performed. In addition, the in vitro 6-OHDA model of neurotoxicity in brain synaptosomes and the in vivo scopolamine (Sco)-induced model of cognitive impairment in rats were also employed. The main antioxidant biomarkers—the levels of lipid peroxidation (LPO) and total glutathione (GSH), as well as activities of superoxide dismutase, catalase, and glutathione peroxidase—were measured in the cortex and hippocampus. **Results**: The results from the in vitro tests demonstrated better ferric-reducing power activity and better neuroprotective capacity of the micellar resveratrol (mRVT), as evidenced by preserved synaptosomal viability and maintained GSH levels in a concentration-dependent manner in 6-OHDA-induced neurotoxicity. Regarding the in vivo results, mRVT (10 µM concentration) was the most effective treatment in supporting recognition memory formation in dementia rats. Further, mRVT demonstrated better LPO protective capacity in the hippocampus and GSH preserving activity in the cortex than the pure drug. **Conclusions**: The incorporation of resveratrol in polymeric micelles could enhance its antioxidant and neuroprotective effects.

## 1. Introduction

Oxidative stress (OS) is increasingly recognized as a crucial factor in the etiology of various neurodegenerative diseases, including age-related Alzheimer’s and Parkinson’s disease. As the longevity of our population increases, these disorders could reach epidemic proportions. Due to its high lipid content, significant oxygen consumption rate, and elevated levels of redox-active metals like iron and copper, the brain is highly susceptible to free radical damage [1,2,3]. Moreover, the intrinsic neurochemical processes, including dopamine metabolism and glutamate-induced excitotoxicity, further enhance this susceptibility. The combination of these factors with an insufficient antioxidant defense system promotes oxidative modifications of essential biomolecules—lipids, proteins, nucleic acids, and carbohydrates—ultimately leading to cellular dysfunction and death [4,5,6,7].

Reactive oxygen species (ROS) play diverse and essential roles in cellular processes, including the regulation of gene expression, cellular differentiation and proliferation, stress response, apoptosis, autophagy, redox signaling, immune function, aging, steroidogenesis, cognitive processes, and thermogenesis [8,9]. Given their involvement in both physiological and pathological mechanisms, maintaining a balance between ROS production and elimination is critical, thus necessitating tight regulation of intracellular ROS levels [10]. Antioxidants counteract the effects of various oxidants, helping to delay, prevent, or inactivate ROS-induced damage at the molecular or tissue level [11]. For instance, diets abundant in natural antioxidants have been linked to a lower risk of various diseases, such as cardiovascular conditions and cancer, and contribute to overall health improvement [12]. In comparison to synthetic antioxidants, naturally occurring compounds are considered safer due to their lower toxicity and are often more effective in exerting protective effects [13].

**Figure 1:**
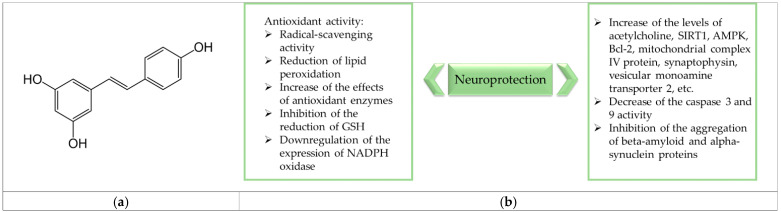
Structure of resveratrol (3,5,4′-trihydroxy-trans-stilbene) (**a**) and main mechanisms of its neuroprotective effect (**b**).

Resveratrol (3,5,40-trihydroxy-trans-stilbene, RVT) is one of the most studied and structurally modulated natural stilbenes [14] (Figure 1). Stilbenes (1,2-diarylethens) represent a very important class of polyphenolic compounds with numerous biological properties (e.g., antioxidant, antiaging, neuroprotective, cardiovascular protective, anti-inflammatory, anticancer) [15,16]. Synthesized as a phytoalexin by plants in response to biotic and abiotic stressors [17], RVT is known as a powerful antioxidant [18]. It has the ability to directly neutralize ROS (hydroxyl radical, hydrogen peroxide, and peroxynitrite) as well as to influence the regulation of the redox systems in general [19]. The antioxidant activity of RVT correlates with an increase in the activity of certain enzymes responsible for reducing oxidative stress, such as heme oxygenase (HO), glutathione peroxidase (GPx), and superoxide dismutase (SOD) [20]. As an activator of SIRT1, it has the ability to modify mitochondrial activity [21]. In addition, there are data that RVT downregulates the expression and activity of NADPH oxidase, thereby inhibiting NADPH oxidase-mediated ROS production, functioning as a gene regulator [22]. Moreover, this naturally occurring stilbene is capable of crossing the blood–brain barrier and demonstrates neuroprotective properties by limiting neuronal degeneration commonly linked to neurodegenerative disorders [23,24]. The mechanisms related to resveratrol’s neuroprotective activity are summarized in Figure 1b.

Despite all the aforementioned properties, RVT has a low potential for clinical use. The main limitations are its hydrophobicity (solubility <0.05 mg/mL), instability, and rapid metabolism in the intestines and liver [25,26]. While being stable in acidic conditions, it degrades exponentially above pH 6.8. Trans-resveratrol undergoes a configurational change to its cis form under light exposure, which lacks significant biological activity [26]. Due to its high lipophilicity, RVT tends to accumulate in cell membranes rather than effectively penetrating them [27,28,29,30,31,32]. These characteristics make resveratrol short-lived and impractical for in vivo use [25,33,34]. There have already been efforts to prepare resveratrol delivery systems capable of overcoming some of these limitations [25,34].

Mixed Pluronic (P123/F127) micelles have been studied as a resveratrol carrier system in our previous research work, where the data indicated that the micellar formulation is a promising platform for therapeutic interventions against neurodegenerative processes [35,36]. Our in vitro findings revealed that the micellar resveratrol (mRVT) provided greater protection than the pure drug at the same concentrations against H_2_O_2_-induced cytotoxicity and oxidative stress in U87MG glioblastoma cells. Additionally, the studies disclosed superior pharmacological effects of the micellar resveratrol compared to the pure drug in an in vivo rat model of experimental dementia.

The aim of the present study was to further evaluate the potential to improve the antioxidant and neuroprotective activity of resveratrol by its incorporation in the micellar system. Thus, we conducted detailed comparative in vitro and in vivo analyses of the antioxidant capacity of resveratrol-loaded Pluronic P123/F127 micelles versus the pure drug. The in vitro antioxidant activity was evaluated by applying DPPH, ABTS, superoxide anion radical-scavenging assays, copper- and ferric-reducing power assays, and iron-induced lipid peroxidation. In addition, the neuroprotective capacity of the micellar resveratrol was examined in 6-hydroxidopamine (6-OHDA)-induced model of neurotoxicity in rat brain synaptosomes. The in vivo antioxidant capacity of RVT and mRVT was compared by determining malonaldehyde (MDA), total glutathione (GSH) levels, superoxide dismutase (SOD), catalase (CAT), and glutathione peroxidase (GPx) activity in the cortex and hippocampus of rats with scopolamine-induced model of cognitive impairment.

## 2. Materials and Methods

### 2.1. Materials

Pluronic^®^ F 127 (PEO_101_PPO_56_PEO_101_) and Pluronic^®^ P 123 (PEO_20_PPO_70_PEO_20_) were provided by BASF (Ludwigshafen, Germany). Scopolamine, trans-resveratrol, thiobarbituric acid, methionine, glutathione reductase, potassium chloride, DPPH (2,2-diphenyl-1-picrylhydrazyl), ABTS (2,2′-azino-bis-(3-ethylbenzothiazoline-6-sulfonic acid)), and Folin reagent were obtained from Sigma-Aldrich Co. (LLC, St. Louis, MO, USA).

### 2.2. Preparation of Resveratrol-Loaded Micelles

The formation of the micelles and the incorporation of resveratrol were simultaneously achieved via the film hydration method. In brief, 40 mg Pluronic P123 and F127 at a ratio of 1:1 (*w*/*w*) were dissolved in methanol. Thereafter, 5 mg of resveratrol was added to the solution. The organic solvent was left to evaporate until the formation of a film. Then the film was redispersed in 4 mL of water, and the dispersion was filtered through a 0.2 µm Nylon filter. UV-Vis spectrophotometric analysis at 306 nm (Thermo Fisher Scientific, Waltham, MA, USA) was applied for the determination of the non-encapsulated amount of resveratrol in the fraction obtained after rinsing the filter with 50% ethanol. The encapsulation efficiency was determined according to the following equation:

EE (%) = (A − B) × 100/A

where A is the total amount of resveratrol, and B is the amount of non-encapsulated resveratrol.

The loading degree of resveratrol in the micellar system was determined according to the following equation:

LD = (A − B)/Volume of the loaded micellar dispersion,

where A is the total amount of resveratrol, and B is the amount of non-encapsulated resveratrol.

The dynamic light scattering (DLS) method was applied for the determination of the mean diameter and polydispersity of the resveratrol-loaded micelles using Zetasizer NanoBrook 90Plus PALS (Brookhaven Instruments Corporation, Holtsville, NY, USA), equipped with a 35-mW red diode laser (λ = 640 nm) at a scattering angle of 90°. The phase analysis light scattering (PALS) method at a scattering angle of 15° was applied for the determination of the zeta potential of the systems.

### 2.3. In Vitro Antioxidant Activity

The radical scavenging capacity of RVT and mRVT was determined and compared by using DPPH, ABTS, and superoxide anion radical scavenging assays. The micellar and pure resveratrol were evaluated at equal concentrations.

#### 2.3.1. DPPH and ABTS Radical Scavenging Activity

The DPPH^●^ free radical scavenging capacity of RVT and mRVT was evaluated and compared by the method of Brand-Williams [37]. Both samples (RVT and mRVT) were added to a methanol solution of DPPH (0.005 mg/mL) in a ratio of 1:1 (*v*/*v*) to reach a final concentration from 8 µg/mL to 500 µg/mL. The mixture was shaken and left for 30 min in the dark at room temperature. The absorbance was red at 517 nm against methanol. The DPPH radical scavenging activity was expressed as a percent inhibition and half-maximal inhibitory concentration (IC_50_). Trolox was used as a reference antioxidant.

The ABTS radical scavenging activity of RVT and mRVT was analyzed and compared according to the method described by Re et al. [38], using a modified version by Raynova et al. [39]. The tested samples were added to the previously formed ABTS radical to reach a final concentration from 0.8 to 54 µg/mL. The scavenging activity of the samples was expressed as a percentage inhibition at maximum absorption of 734 nm. The IC_50_ was also determined and compared with the reference standard Trolox.

#### 2.3.2. Superoxide Anion Scavenging Assay (NBT)

The method of Beauchamp and Fridovich [40] for the generation of superoxide anion radicals (^●^O_2_^−^) was applied. The samples were added to the reaction mixture (0.05 M KPO_4_, pH 7.8, riboflavin, methionine, nitro-blue tetrazolium (NBT), potassium cyanide) to reach a final concentration ranging from 1 µg/mL to 33 µg/mL. The reduction of NBT by ^●^O_2_^−^ to an insoluble blue formazan product was measured. The antioxidant effects of RVT and mRVT were detected at 560 nm, and expressed as a percentage of the control (100%).

#### 2.3.3. Ferric-Reducing Power Assay (FRAP)

The method of Benzie and Strain [41] was conducted. After preparing the working mixture (acetate buffer, TPTZ (2,4,6-tri(2-pyridyl)-s-triazine), and ferric chloride (FeCl_3_)), the tested compounds RTV and mRVT were added to the FRAP generating system at a final concentration ranging from 0.52 µg/mL to 33 µg/mL. Their ferric-reducing capacities were subsequently assessed and compared by measuring the absorbance at 593 nm against a blank sample. A stock solution of 1 mM Trolox was diluted to 500, 250, 125, 62.5, 31.25, 15.6, 7.8, 3.9, and 2 µM concentrations, which were suitable for the preparation of a standard curve. The results were expressed as µM Trolox equivalent (TE) using the calibration curve of Trolox.

#### 2.3.4. Copper-Reducing Power Assay (CUPRAC)

The copper-reducing antioxidant capacity (CUPRAC) was determined following the method described by Apak et al. [42]. RVT and mRVT (concentrations ranging from 2 µg/mL to 133 µg/mL) were added to the reaction mixture containing ammonium acetate buffer, copper (II) chloride solution, and neocuproine. The samples were mixed and incubated at 50 °C for 20 min. The absorbance was measured at 450 nm against a blank sample containing only the solvent and reaction mixture. The results were expressed as µM Trolox equivalents.

#### 2.3.5. Iron-Induced Lipid Peroxidation (TBA-Test)

The method of Hunter et al. [43], based on the reaction of thiobarbituric acid with final products of lipid oxidation and formation of malondialdehyde (MDA), was applied. RVT and mRVT (concentrations from 0.02 µg/mL to 10 µg/mL) were dripped in rat’s brain homogenate (mg/protein/mL) and then incubated at 37 °C for 30 min in the presence of 10 mM FeCl_3_ and 10 mM ascorbic acid. The next step was to add a mixture of THO:HCl:TBA (2:1:2 ratio) to the samples and to boil them for 15 min in a water bath. The samples were cooled down and centrifuged at 3000 rpm. The absorbance was then measured at 532 nm against a blank sample. The results were expressed as a percentage inhibition of lipid peroxidation, based on the MDA content relative to the control (set at 100%), as well as IC_50_ values. The antioxidant properties of pure resveratrol and its encapsulated form were compared with reference Trolox.

### 2.4. In Vivo Antioxidant Activity

#### 2.4.1. Animals

Male Wistar rats (250–280 g) were obtained from Erboj (Slivniza, Sofia, Bulgaria). The animals were housed four per cage under standard laboratory conditions (25 ± 3 °C, 12-h light/dark cycle) with free access to food and tap water. Experimental procedures were initiated after a five-day acclimatization period. All animal work was performed in compliance with national regulations and approved by the Bulgarian Food Safety Agency (Approval No. 397/23.05.24).

#### 2.4.2. Experimental Design

The male Wistar rats (n = 6 in each group) were randomly divided into 6 experimental groups and injected intraperitoneally (i.p) for 11 consecutive days as follows:

- Control (0.9% NaCl, i.p);
- Sco (scopolamine 2 mg/kg, i.p);
- Sco + RVT 5 (5 mg/kg RVT, i.p);
- Sco + RVT 10 (10 mg/kg RVT, i.p);
- Sco + mRTV 5 (5 mg/kg RVT, i.p);
- Sco + mRTV 10 (10 mg/kg RVT, i.p).

**Figure 2:**
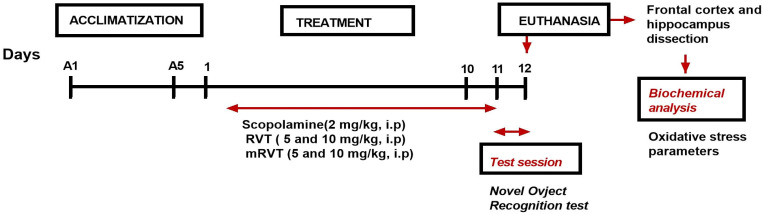
Experimental design.

RVT and mRVT in the two doses (5 and 10 mg/kg) [35,44] were applied 1 h before Sco (Figure 2).

For verification of the model, the animals from all groups were subjected to the novel object recognition (NOR) behavioral test 12 days after the first Sco treatment. One hour after the tests, the animals were euthanized.

#### 2.4.3. Novel Object Recognition Test (NOR)

The novel object recognition test, initially described by Ennaceur and Delacour [45], is based on the tendency of healthy rodents to interact with novel surroundings [46]. The NOR test was conducted in a white-painted square box (50 × 50 × 50 cm) placed in a dimly lit room. To avoid odor trails, the apparatus and objects were cleaned with water between trials. The objects varied in shape, color, and texture.

Animals were placed in the experimental room at least 30 min before testing. The testing procedure took two days. On Day 1, the habituation session allowed the rats to explore two identical objects placed inside the arena for 3 min. On Day 2, the acquisition and test trials were performed. During the acquisition trial, 24 h after habituation, each animal was placed in the arena with two identical, familiar objects for 4 min. In the subsequent test trial, one of the familiar objects was replaced with a novel one. The animals were then placed back in the arena for 3 min, and the total time spent exploring each object was recorded.

Recognition memory was evaluated using a discrimination index (DI), calculated for each animal as follows: (N/N+F) × 100, where N is the time spent with the novel object, and F is the time spent with the familiar object.

#### 2.4.4. Tissue Preparation

The levels of lipid peroxidation (LPO) and total glutathione (GSH) were measured in the post-nuclear fraction obtained after homogenization of the cortex and hippocampus in 0.15 M KCl and centrifugation at 3000× *g* for 10 min at 4 °C. The activities of antioxidant enzymes were assessed in the post-mitochondrial fraction, prepared by further centrifugation of the post-nuclear fraction at 12,000× *g* for 20 min at 4 °C.

#### 2.4.5. Oxidative Stress Parameters

Lipid peroxidation levels, as well as catalase and glutathione peroxidase activities, were quantified spectrophotometrically using commercial assay kits in accordance with the manufacturer’s protocols: Lipid Peroxidation (MDA) Assay Kit (MAK085), Catalase Assay Kit (CAT100), and Glutathione Peroxidase Cellular Activity Assay (CGP1) (Sigma-Aldrich, St. Louis, MO, USA).

The total glutathione level was measured by the method of Rahman et al. [47]. SOD activity was determined according to Peskin and Winterbourn [48]. The protein content was measured by the method of Lowry et al. [49] and was determined using a calibration curve obtained with bovine serum albumin (Pentex, Petaluma, CA, USA).

### 2.5. Neuroprotective Capacity

#### 2.5.1. Synaptosomal Viability Assay

The synaptosomes used in this study were prepared from rat brains by applying the reported protocol [50]. First, the brains were homogenized in cold buffer (pH 7.4), consisting of 5 mM HEPES and 0.32 M sucrose. After that, centrifugation of the homogenate was applied twice (1000× *g*, 5 min, 4 °C), followed by collection of the supernatant and centrifugation 3 times (10,000× *g*, 20 min, 4 °C). Then, the obtained pellet was resuspended in the ice-cold buffer mentioned above. A Percoll reagent was used to isolate the synaptosomes that were further incubated in a buffer consisting of 290 mM NaCl, 0.95 mM MgCl_2_x6H_2_O, 10 mM KCl, 2.4 mM CaCl_2_xH_2_O, 2.1 mM NaH_2_PO_4_, 44 mM HEPES, and 13 mM D-glucose. The incubations took place in a 5% CO_2_ + 95% O_2_ atmosphere. The method of Lowry et al. [49], which utilized a serum albumin as a standard, was applied for the determination of the content of synaptosomal protein.

The isolated rat synaptosomes were treated with 6-hydroxydopamnine (6-OHDA) (150 µM), pure and micellar resveratrol (0.1–20 µg/mL) for 1 h. After the simultaneous incubation of the samples with RVT or mRVT and 6-ODHA, centrifugation of the synaptosomes for 1 min at 15,000× *g* was performed. Thereafter, a MTT solution (60 µL) was added to each well with the ‘washed’ synaptosomes, followed by incubation of the plates at 37 °C for 10 min. Then the samples were centrifuged at 15,000× *g* for 1 min; the excess liquid was removed, and a DMSO solution was applied in order to dissolve the formed formazan crystals. The crystals were dissolved, and the amount of formazan was measured via a spectrophotometer at λ = 580 nm [51].

#### 2.5.2. GSH Determination in Isolated Brain Synaptosomes

After the incubation of RVT or mRVT with 6-OHDA, the synaptosomes were centrifuged at 4000× *g* for 3 min. The supernatant was removed, and the pellet was treated with 5% trichloroacetic acid, left for 10 min on ice, and centrifuged at 8000× *g* for 10 min (2 °C). The supernatant was taken for GSH determination and frozen at −20 °C. The levels of glutathione (GSH) were determined with the Ellman reagent (DTNB), which forms colored complexes with the thiol groups at pH = 8.0 (neutralized with 5N NaOH) with maximum absorbance at 412 nm [52].

### 2.6. Statistical Analysis

All results are presented as mean ± standard error of the mean (SEM). Data from the in vivo experiments were analyzed using one-way analysis of variance (ANOVA) followed by Tukey’s post hoc test in GraphPad Prism 9.0. The data from the in vitro neurotoxicity model were evaluated using the statistical software MEDCALC 23.3.7 and analyzed using the non-parametric Mann–Whitney test. Free radical scavenging activity was analyzed using the Student’s *t*-test. Differences were considered statistically significant at *p* < 0.05.

## 3. Results

### 3.1. Characterization of Resveratrol-Loaded Micelles

**Figure 3:**
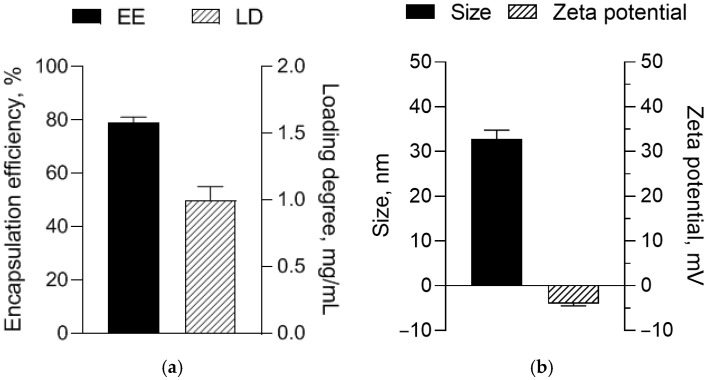
Encapsulation efficiency and loading degree of resveratrol in the micelles (**a**) and size and zeta potential of the loaded micelles (**b**).

The micellar system was characterized with 79% encapsulation efficiency and a loading degree of 1 mg resveratrol/mL micellar dispersion (Figure 3a). Moreover, the DLS analysis (Figure 3b) revealed a small mean diameter of the micelles (32.8 nm), a zeta potential close to zero (−4 mV), and a narrow size distribution (PDI = 0.278).

### 3.2. In Vitro Antioxidant Activity

**Figure 4:**
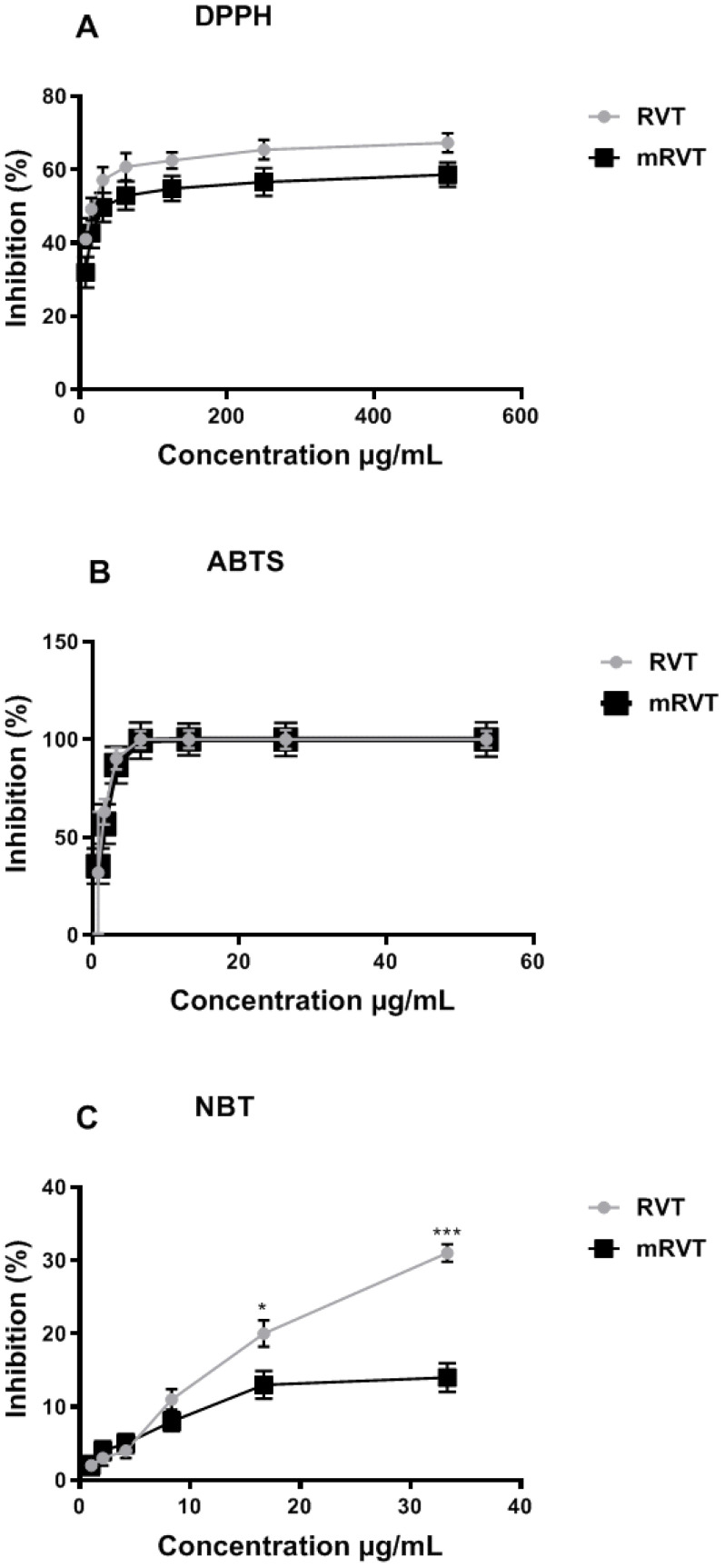
Free radical scavenging activity of RVT and mRVT against DPPH (**A**), ABTS (**B**), and NBT (**C**), presented as percentage inhibition. Data are in triplicate, expressed as the mean ± SEM. Data analysis was performed using Student’s *t*-test. Significance * *p* < 0.05, *** *p* < 0.001.

DPPH is a stable free radical that is used as a reagent to evaluate the free radical scavenging activity of antioxidants [53,54]. The radical scavenging activity of RVT and mRVT against DPPH was measured in a concentration range from 8 to 500 µg/mL (Figure 4A). At the minimal tested concentration, RVT inhibited DPPH radical by 41.03%, and mRVT by 31.95%. The maximal scavenging activity of RVT and mRVT was observed at a concentration of 500 µg/mL, with RVT exhibiting higher activity compared to mRVT (67.32% and 58.6%, respectively). The IC_50_ for the tested and standard compounds decreased in the following order: RVT (IC_50_ = 21.9 µg/mL) > mRVT (IC_50_ = 34.19 µg/mL) > Trolox (IC_50_ = 41.7 µg/mL) (Table 1), demonstrating higher scavenging activity for pure and micellar resveratrol.

As seen in Figure 4B, RVT and mRVT are effective ABTS radical scavengers in a concentration-dependent manner (from 0.8 to 54 µg/mL). The maximal inhibition for both forms of resveratrol was at a concentration of 6.6 µg/mL (100% for RVT and 99.39% for mRVT). The IC_50_ values of both samples were similar: 1.23 µg/mL for RVT and 1.30 µg/mL for mRVT (Table 2). Compared to Trolox (IC_50_ = 8.6 µg/mL), the pure and encapsulated resveratrol manifested approx. seven times better effect (Table 1).

The superoxide radical scavenging activity of RVT and mRVT in a concentration range from 1 to 33 µg/mL is presented in Figure 4C. Our results showed that the pure and micellar forms of resveratrol demonstrated the same activity up to 4.2 µg/mL concentration. With increasing concentration, the percentage of ^●^O_2_^−^ inhibition by RVT was significantly higher than that observed for mRVT. The maximal inhibition of ^●^O_2_^−^ by RVT was 31%, which was nearly two times higher than that of mRVT (14%) at a concentration of 33 µg/mL.

The ferric-reducing antioxidant power activity of RVT and mRVT was estimated in a final concentration range from 0.5 to 33 µg/mL (Table 2). The results were calculated as µM Trolox equivalent (ET) and showed that mRVT possessed better ferric reducing capacity than RVT in the tested concentrations. The minimal reducing ability was observed at 0.5 µg/mL, namely 3.02 µM ET for mRVT and 1.77 µM ET for RVT. The maximal FRAP was detected at 33 µg/mL, namely 8.57 µM ET for mRVT and 6.70 µM ET for RVT.

The cupric reducing antioxidant ability of RVT and mRVT was tested in a final concentration range from 2 to 133 µg/mL (Table 2). The free resveratrol showed higher CUPRAC capacity at all tested concentrations compared to the micellar form. At the lower concentration (2.1 µg/mL), the cupric reducing antioxidant ability for RVT was 21.33 µM ET, whereas for mRVT it was 6.20 µM ET. At a concentration of 66.7 µg/mL, the tested substances showed maximal CUPRAC, namely 260.87 µM ET for RVT and 240.59 µM ET for mRVT. Above this concentration, a rise in the CUPRAC of the tested substances was not observed.

**Figure 5:**
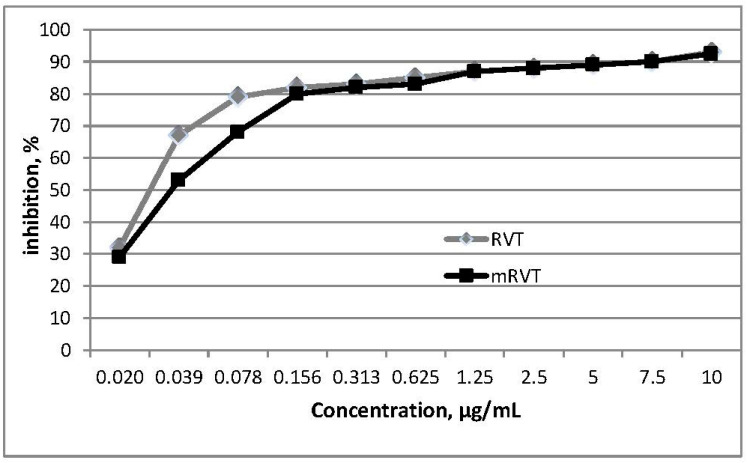
In vitro effects of pure (RVT) and micellar (mRVT) resveratrol on the levels of iron-induced lipid peroxidation in rat’s brain. Data are expressed as the mean ± SD (n = 3).

For the iron-induced lipid peroxidation, pure and micellar resveratrol were tested in a final concentration range from 0.02 to 10 µg/mL in a solution containing brain homogenate, iron, and ascorbic acid. RVT and mRVT displayed significantly powerful antioxidant capacity against the lipid peroxidation products. At the lowest tested concentration of 0.02 µg/mL, about 30% antioxidant activity was detected, while at 0.04 µg/mL, it was 50%. At the concentration of 0.156 µg/mL, there was about 80% inhibition of LPO. Regardless of the different solubility, both samples demonstrated similar, but very strong, antioxidant properties (Figure 5). The IC_50_ for the samples and Trolox decreased in the following order: RVT (IC_50_ = 0.021 µg/mL) > mRVT (IC_50_ = 0.036 µg/mL) > Trolox (IC_50_ = 37.6 µg/mL) (Table 1).

### 3.3. In Vivo Antioxidant Activity

#### 3.3.1. Effect of RVT and mRVT on Recognition Memory of Rats with Scopolamine-Induced Memory Impairment (Novel Object Recognition Test)

**Figure 6:**
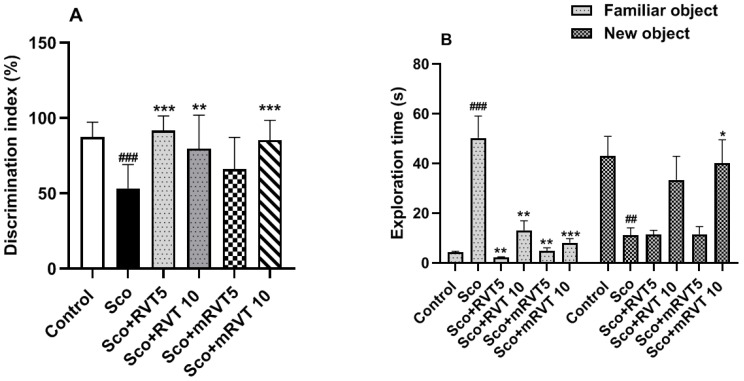
Effect of RVT and mRVT (5 and 10 mg/kg) on discrimination index (**A**) and exploration time (**B**) of rats with Sco-induced memory deficit in the novel object recognition test. Each column represents mean ± SEM of 6 animals. Significance vs. control: ^##^ *p* < 0.01, ^###^ *p* < 0.001, significance vs. scopolamine: * *p* < 0.05, ** *p* < 0.01, *** *p* < 0.001.

On the 12th day of the experiment, the animals were tested for their ability to discriminate a novel object from familiar ones using the novel object recognition test (Figure 6A,B). Our data showed that the Sco-administered rats spent more time with the familiar object, indicating poor memory and an inability to differentiate the novel object from familiar ones (Figure 6B). The discrimination index in this group was reduced by 39% (*p* < 0.001, n = 6) compared to the control group (Figure 6A).

The effect of Sco was significantly reversed after RVT/mRVT (5 and 10 mg/kg) administration. As shown in Figure 6A, DI in RVT 5, RVT 10, mRVT 5 and mRVT 10 treated dementia rats was increased, compared to the model (Sco) group by 72% (*p* < 0.001, n = 6), 50% (*p* < 0.01, n = 6), 24% (ns) and 65% (*p* < 0.001, n = 6), respectively. Our results highlighted the animals treated with mRVT 10 as the group that spent the longest time with the novel object compared to all other groups (Figure 6B). Specifically, the mean time spent with the novel object was 43 s for the control group, 33 s for the RVT 10 group, and 40 s for the mRVT 10 group. The time spent with the novel object in the RVT 5 and mRVT 5 groups was comparable to that of the Sco-treated rats, namely approximately 11 s.

#### 3.3.2. Effect of RVT and mRVT on LPO and GSH Levels in the Cortex and Hippocampus of Rats with Scopolamine-Induced Memory Impairment

**Figure 7:**
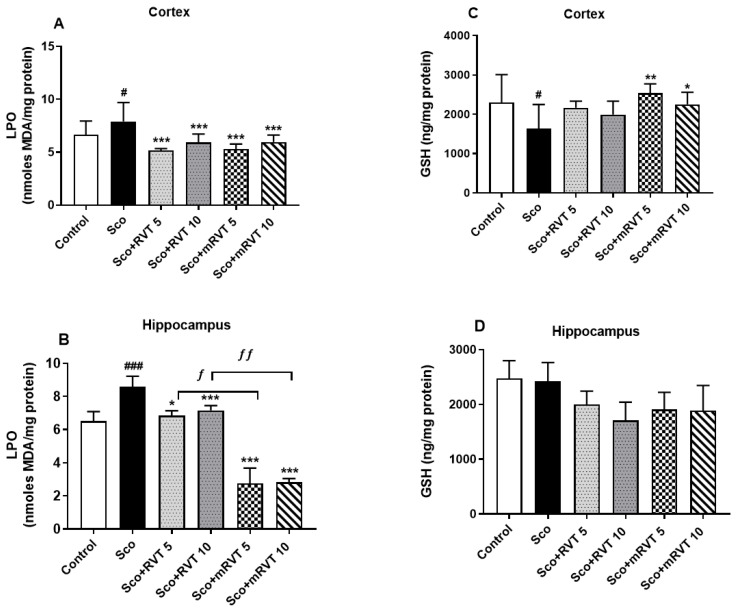
Effects of pure (RVT) and micellar (mRVT) resveratrol on the levels of LPO and GSH in cortex (**A**,**C**) and hippocampus (**B**,**D**) in rats with scopolamine-induced dementia. Data are expressed as the mean ± SEM of 6 animals. Significance vs. control: ^#^ *p* < 0.05, ^###^ *p* < 0.001; significance vs. scopolamine: * *p* < 0.05, ** *p* < 0.01, *** *p* < 0.001; significance between RVT and mRVT-treated groups: ^ƒ^ *p* < 0.05, ^ƒƒ^ *p* < 0.01.

In the model (Sco) group, 11 consecutive days of Sco-treatment increased the lipid peroxidation levels by 19% (*p* < 0.05, n = 6) in the cortex (Figure 7A) and by 32% (*p* < 0.001, n = 6) in the hippocampus (Figure 7B) compared to the control. The total GSH content was significantly affected only in the cortex, where a decrease by 28% (*p* < 0.05, n = 6) was observed (Figure 7C). RVT and mRVT treatment reversed the Sco-induced changes in the observed oxidative stress markers. Micellar resveratrol exerted a statistically significant and more pronounced effect than pure RVT on LPO levels in the hippocampus and on total GSH in the cortex.

#### 3.3.3. Effect of RVT and mRVT on SOD, CAT, and GPx Activity in the Cortex and Hippocampus of Rats with Scopolamine-Induced Memory Impairment

**Figure 8:**
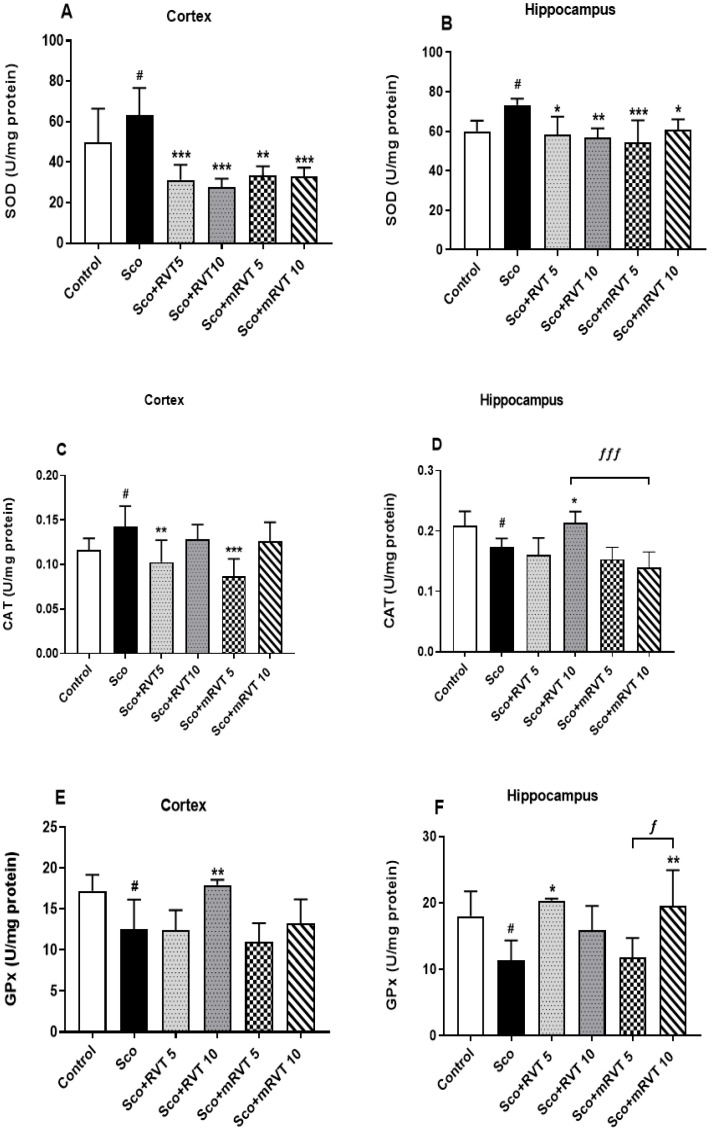
Effects of pure (RVT) and micellar (mRVT) resveratrol on SOD, CAT and GPx activity in cortex (**A**,**C**,**E**) and hippocampus (**B**,**D**,**F**) in rats with scopolamine-induced dementia. Data are expressed as the mean ± SEM of 6 animals. Significance vs. control: ^#^ *p* < 0.05; significance vs. scopolamine: * *p* < 0.05, ** *p* < 0.01, *** *p* < 0.001; significance between RVT and mRVT-treated groups: ^ƒ^ *p* < 0.05, ^ƒƒƒ^ *p* < 0.001.

In the model (Sco) group, 11 consecutive days of Sco treatment significantly altered SOD, CAT, and GPx enzyme activity in both brain structures (Figure 8). The SOD activity was increased by 27% (*p* < 0.05, n = 6) in the cortex and by 22% (*p* < 0.05, n = 6) in the hippocampus; CAT activity was increased by 17% (*p* < 0.05, n = 6) in the cortex and decreased by 15% (*p* < 0.05, n = 6) in the hippocampus; GPx activity was decreased by 27% (*p* < 0.05, n = 6) in the cortex and by 24% (*p* < 0.05, n = 6) in the hippocampus. The treatment with RVT and mRVT ameliorated these Sco-induced changes in the enzyme activity with similar effectiveness.

### 3.4. Neuroprotective Capacity in a Synaptosomal Model of Neurotoxicity

The potential neuroprotective effect of RVT and mRVT was also assessed in a synaptosomal model of neurotoxicity. First, the potential cytotoxic effect of RVT and mRVT on the rat brain synaptosomes was defined by evaluating the synaptosomal viability at concentrations of 0.1–20 µg/mL. The results showed that RVT and mRVT did not exhibit any statistically significant neurotoxic effects in comparison with the control (non-treated brain synaptosomes), indicating the safety of the treatment (not shown).

**Figure 9:**
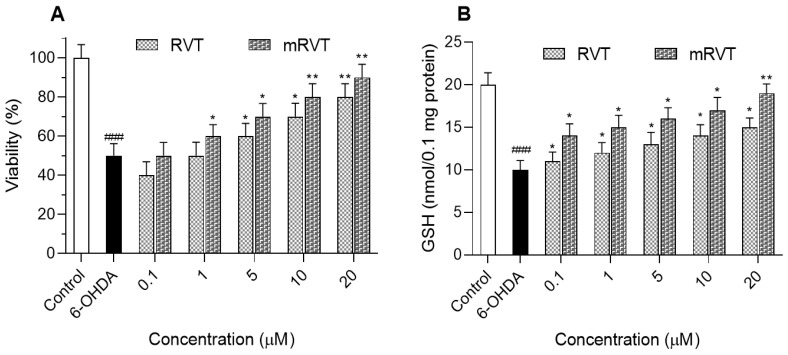
Effects of pure (RVT) and micellar (mRVT) resveratrol on viability (**A**) and GSH level (**B**) in 6-OHDA-induced neurotoxicity in rat brain synaptosomes. * *p* < 0.05; ** *p* < 0.01 vs. 6-OHDA group; ^###^ *p* < 0.001 vs. control.

The treatment with 150 μM 6-OHDA showed a statistically significant decrease in the synaptosomal viability compared to the control (Figure 9). In the in vitro model of 6-OHDA-induced neurotoxicity, there was a concentration-dependent protective effect of the micellar resveratrol. An increase of 10, 20, 30, and 40% of the synaptosomal viability was observed at 1, 5, 10, and 20 µg/mL concentration of the micellar resveratrol, respectively. For comparison, the treatment with the pure resveratrol achieved a lower degree of protection (0, 10, 20, and 30%, respectively). The ability of the micelles to preserve the levels of GSH was evaluated, too. There was also a concentration-dependent protective effect. The micellar drug induced 20, 25, 30, 35, and 45% protection of the GSH levels at concentrations of 0.1, 1, 5, 10, and 20 µg/mL in comparison to the toxic agent (6-OHDA) (Figure 9B). At the same time, the effect of the non-encapsulated resveratrol was less pronounced.

## 4. Discussion

As a molecule with antioxidant capacity, RVT is extensively researched regarding its effects against oxidative stress-related disorders. However, its unfavorable biopharmaceutical properties limit its in vivo use. In a previous study, our group showed that the encapsulation of RVT in Pluronic micelles for neuronal delivery enhanced its biological activity in vivo [35]. We also revealed that mRVT possesses stronger intracellular antioxidant activity in low concentrations (1 and 3 μM) compared to its pure form in the U87MG glioblastoma cell line with H_2_O_2_-induced cytotoxicity [36].

In the present study, the antioxidant activity of pure RVT (hydroethanolic solution) and mRVT (micellar dispersion) was evaluated and compared in vitro and in vivo. The in vitro antioxidant activities of RVT and mRVT were compared using common bioanalytical methodologies, including DPPH, ABTS, NBT, FRAP, and CUPRAC assays, as well as by conducting an assay evaluating their ability to mitigate oxidative damage under conditions of induced lipid peroxidation. In addition, the antioxidant activity of RVT and mRVT was compared to that of Trolox, which is usually used as a reference standard. Our results demonstrated higher antioxidant activity of RVT in comparison with the standard. This correlates with previous reports indicating higher DPPH, ABTS, FRAP, and CUPRAC activity of RVT [54,55,56,57]. Moreover, the applied in vitro rapid antioxidant screening methods demonstrated that mRVT exhibited greater ferric ion-reducing activity at all tested concentrations (0.5–33 µg/mL), compared to the pure drug. In the DPPH, ABTS, CUPRAC, and induced lipid peroxidation assays, the in vitro antioxidant activity of mRVT was comparable to that of RVT. This finding is important since it is well known that the presence of free redox ions in biological systems, mainly Fe (II) and Cu (I), is a potential source of oxidative stress because they are involved in Fenton’s reaction, which results in the formation of the highly reactive and destructive ^●^OH radical [58]. Furthermore, Aβ-toxicity, characteristic of AD, is associated with the presence of redox metals. The deposition of amyloid plaques is related to the chelation of amyloid β peptide with ions of the transition metals Cu^2+^, Zn^2+^, and Fe^3+^ [59].

Considering the fact that the encapsulation of RVT maintained its in vitro antioxidant activity, the scopolamine-induced memory impairment model on male Wistar rats was used to evaluate the in vivo antioxidant potential of the micellar system. Scopolamine is an alkaloid, a nonselective muscarinic receptor antagonist, with a reversible action that specifically affects the cholinergic neurons. Its application is associated with the generation of oxidative stress, evident as an elevated lipid peroxidation level, reduction in antioxidant levels in the brain, and altered activity of the endogenous enzymatic antioxidant system [35,60,61,62]. These effects impact hippocampal and cortical functions, leading to memory and cognitive impairments, resembling symptoms of Alzheimer’s-type dementia [63]. The in vivo memory protective effect of mRVT was tested via the novel object recognition test. This test has been widely used to study memory functions in rodents, emphasizing the connection between novelty and animal behavior [63]. According to the fundamental principle of the classical novel object recognition test, a preference for the novel object indicates that the familiar object is retained in the animal’s memory [64]. The task involves both exploratory behavior and memory retention components, requiring the animal to sufficiently explore the familiar object during the pretest phase and to distinguish it from the novel object during the test phase [65]. Our results revealed that the Sco-treatment decreased the time that animals spent with the novel object compared to the control. This behavior resulted in a reduced discrimination index, an indication of the recognition memory impairment. Both mRVT 10 and RVT 10 treatments restored the exploration time of the novel object in the dementia animals to control levels. However, the effect was statistically significant only for mRVT 10. Neither mRVT 5 nor RVT 5 reversed the scopolamine-induced reduction in the exploration time of the novel object. These results highlighted mRVT 10 as the most effective in sustaining the recognition memory formation in the scopolamine-induced dementia rat model. This is in accordance with our previous research where mRVT at a dose of 10 mg/kg was selected over pure RVT (5 mg/kg and 10 mg/kg) and mRVT (5 mg/kg) treatments due to its better ability to ameliorate behavioral and biochemical complications in a scopolamine-induced experimental model of dementia in rats [35].

Moreover, the levels of five main oxidative stress markers, encompassing enzyme and non-enzyme defense systems, namely LPO, GSH, SOD, CAT, and CPx enzymes, were evaluated. The destructive nature of lipid peroxidation and its chain products is of vital importance for biological membranes [66]. The pivotal role of the cell membrane is to serve as a barrier from exterior invasion, protecting the cell from damage. The alteration of the lipid cellular integrity is one of the main pathophysiological mechanisms, leading to neurodegenerative disorders [67]. Lipid peroxidation byproducts and the iron ions involved in the reactions (iron-mediated lipid peroxidation) are one of the main mechanisms leading to ferroptosis [68]. The levels of MDA were measured as an equivalent for the cells membrane disruption in the cortex and the hippocampus of the rats. Sco-application increased LPO levels in both brain structures, whereas both the pure and the micellar resveratrol reversed this effect. This is in agreement with some data in the literature, confirming that in some neurodegenerative disorders, such as Alzheimer’s, Parkinson’s and Huntington’s diseases, resveratrol application significantly suppresses lipid peroxidation, thus showing the neuroprotective effect [69,70,71]. Resveratrol protects polyunsaturated fatty acids from oxidation, delaying and forbidding lipid peroxidation processes via breaking down the chain reactions by removing the produced lipid peroxide products [72,73]. Our results clearly demonstrated that the micellar form of resveratrol exerted the strongest antioxidant and neuroprotective effects in the hippocampus. No significant difference was observed between the effects of mRVT at doses of 5 and 10 mg/kg.

Glutathione (GSH) is a key endogenous antioxidant that plays a crucial role in maintaining the redox balance and supporting various cellular functions in the nervous system. Mitochondrial dysfunction, reflecting on GSH depletion, can evoke abnormally low glutathione concentrations. This is associated with increased oxidative stress and neuronal damage [73]. For instance, Mandal et al. [74] demonstrated significantly reduced glutathione concentrations in the cortex and hippocampus in neurodegenerative models. Moreover, resveratrol treatment has been shown to elevate total GSH levels, thereby exerting neuroprotective effects in conditions such as Alzheimer’s disease and Parkinson’s disease [75]. Our results showed that the treatments affected statistically significant GSH levels only in the cortex. Sco-administration decreased GSH levels, whereas only mRVT, at both tested doses, restored GSH levels to control values.

The most common antioxidant enzymes, such as SOD, CAT, and GPx, utilize their specific substrates to reduce the production of oxidants that cause direct damage to macromolecules [76,77]. Our results showed that Sco had a negative impact on the activity of all three enzymes, whereas the pure and the micellar resveratrol ameliorated this effect. This was in line with previously reported data that polyphenols such as RVT have the ability to elevate the expression of main antioxidant enzymes such as superoxide dismutase, catalase, and glutathione peroxidase for removing the generated ROS [78]. Our tests revealed that the incorporation of resveratrol in micelles retained its activity.

The neuroprotective potential of the pure and micellar resveratrol was also evaluated in an in vitro 6-OHDA-induced model of neurotoxicity in rat brain synaptosomes. Moreover, 6-OHDA is a redox cycling dopamine analogue that causes oxidative stress by inhibition of mitochondrial chain complex I, leading to overproduction of ROS as hydroxyl radicals, superoxide, and hydrogen peroxide [79]. First, the pure and micellar resveratrol did not show any significant neurotoxic effects on brain synaptosomes in the range of 0.1–20 µg/mL. There were no statistically significant changes in the biomarkers, which characterize the functional and metabolic status of the synaptosomes, namely synaptosomal viability and level of reduced glutathione (GSH). These results are consistent with previously reported data for the lack of toxicity of empty micelles, RVT, and mRVT on the U87MG glioblastoma cell line [36]. More importantly, in the present study, we found that the formulation of resveratrol in the micelles led to an enhancement of its neuroprotective activity against 6-OHDA-induced toxicity even at the lowest concentration (0.1 µM), where the pure drug was not active. The results showed a concentration-dependent protection of synaptosomes, as well as a maintenance of the GSH levels. Similar dose-dependent neuroprotective effects of resveratrol were also observed by other study groups.

Despite the promising results regarding the antioxidant and neuroprotective activity of micellar resveratrol, we acknowledge that the present study has several limitations that warrant further investigation. Thus, future studies should address histopathological analyses of key brain regions (e.g., frontal cortex and hippocampus) as well as systemic or brain toxicity.

## 5. Conclusions

Our results revealed that the micellar form of resveratrol retains its antioxidant effects and, in some of the models, leads to better antioxidant activity than the pure drug. In vitro, the micellar resveratrol showed higher ferric reducing power activity as well as stronger neuroprotective capacity in 6-OHDA-induced neurotoxicity in brain synaptosomes. The results from the in vivo experiments highlighted the 10 mg/kg micellar resveratrol as the most effective in sustaining the recognition memory formation in the scopolamine-induced dementia rat model. In addition, the micellar form of resveratrol demonstrated better LPO protective capacity in the hippocampus and GSH preserving activity in the cortex than the pure drug. Thus, the Pluronic micelles loaded with resveratrol could be considered as a candidate for future clinical studies in neurodegenerative diseases.

## Figures and Tables

**Table 1:** IC_50_ concentrations of RVT, mRVT, and Trolox in DPPH, ABTS and TBA assays.

Sample	DPPH	ABTS	TBA
RVT	21.9 ± 0.51	1.23 ± 0.02	0.021 ± 0.001
mRVT	34.19 ± 0.9	1.3 ± 0.016	0.036 ± 0.002
Trolox	41.7 ± 1.32	8.6 ± 0.79	37.6 ± 1.32

**Table 2:** FRAP and CUPPRAC assay of RVT and mRVT.

Ferric-Reducing Power Assay of RVT and mRVT			Copper-Reducing Power Assay of RVT and mRVT		
Final Concentration (µg/mL)	Expressed as Trolox Equivalent (ET), µM		Final Concentration (µg/mL)	Expressed as Trolox Equivalent (ET), µM	
	RVT	mRVT		RVT	mRVT
0.52	1.77 ± 0.01	3.02 ± 0.03	2.08	21.34 ± 1.23	6.20 ± 0.05
1.04	1.78 ± 0.2	3.68 ± 0.13	4.16	49.47 ± 2.22	30.32 ± 1.37
2.08	2.71 ± 0.05	5.33 ± 0.06	8.33	82.64 ± 3.45	59.75 ± 2.76
4.17	3.76 ± 0.07	6.67 ± 0.23	16.67	143.77 ± 4.25	106.94 ± 5.12
8.33	5.20 ± 0.21	7.80 ± 0.32	33.33	230.68 ± 2.91	184.98 ± 3.46
16.67	6.09 ± 0.07	8.31 ± 0.16	66.67	260.87 ± 4.35	240.59 ± 3.86
33.33	6.70 ± 0.04	8.57 ± 0.18	133.33	264.33 ± 3.21	241.00 ± 3.01

Data are presented as µM Trolox equivalent, mean ± SD (n = 3).

## Data Availability

The original contributions presented in this study are included in the article. Further inquiries can be directed to the corresponding author.

## Abbreviations

The following abbreviations are used in this manuscript:

ABTS	2,2′-azino-bis-(3-ethylbenzothiazoline-6-sulfonic acid
AChE	Acetylcholineesterase
AD	Alzheimer’s disease
CAT	Catalase
CUPPRAC	Cupric reducing antioxidant capacity
DPPH	2,2-diphenyl-1-picrylhydrazyl
FRAP	Ferric reducing antioxidant power
GPx	Glutathione peroxidase
GSH	Glutathione
IC_50_	Half maximal inhibitory concentration
i.p.	Intraperitoneal
LPO	Lipid peroxidation
MDA	Malonedialdehyde
NADPH	Nicotine adenine dinucleotide phosphate hydrate
ROS	Reactive oxygen species
RVT	Resveratrol
mRVT	Micellar resveratrol
OS	Oxidative stress
Sco	Scopolamine
SOD	Superoxide dismutase
TE	Trolox equivalent
TBA	Thiobarbituric acid
6-OHDA	6-hydroxydopamine hydrobromide
